# CDC’s Sodium Reduction in Communities Program: Evaluating Differential Effects in Food Service Settings, 2013–2016

**DOI:** 10.5888/pcd17.190446

**Published:** 2020-07-30

**Authors:** Julia Jordan, Hadley Hickner, John Whitehill, Benjamin Yarnoff

**Affiliations:** 1 IHRC, Inc, Atlanta, Georgia; 2 Centers for Disease Control and Prevention, Division for Heart Disease and Stroke Prevention, Atlanta, Georgia; 3 RTI International, Public Health Economics Program, Research Triangle Park, North Carolina

## Abstract

High sodium intake can lead to hypertension and increase the risk for heart disease and stroke; however, research is lacking on the effectiveness of community-based sodium reduction programs. From 2013 through 2016, the Centers for Disease Control and Prevention (CDC) funded 10 state and local health departments to implement sodium reduction strategies across diverse institutional food settings. Strategies of the Sodium Reduction in Communities Program (SRCP) are implementing food service guidelines, making menu modifications, enabling purchase of reduced-sodium foods, and providing consumer information. CDC aggregated awardee-reported performance measures to evaluate progress in increasing the access, availability, and purchase of reduced sodium foods. Evaluation results of the SRCP show the potential differential effects of sodium reduction strategies in a community setting and support the need for additional community-level efforts in this emerging area of public health.

**Summary**

**What is already known on this topic?**

Most people in the United States exceed the recommended daily sodium intake from the sodium already in processed and restaurant foods, whether or not they pick up a saltshaker.

**What is added by this report?**

From 2013 through 2016, CDC’s Sodium Reduction in Communities Program demonstrated the extent to which the program’s strategies had successfully increased access, availability, and purchases of reduced sodium foods. The program also demonstrated the differential effects of sodium reduction strategies in food service settings.

**What are the implications for public health practice?**

Tailored approaches that are based on a community’s available resources, stage of readiness, and food service staff’s level of engagement can address some of a strategy’s differential effects in food service settings.

## Introduction

Excessive sodium intake is associated with increased risk of high blood pressure, coronary heart disease, and stroke (1–3). Nearly 85% of US adults and children currently exceed the 2015 to 2020 Dietary Guidelines for Americans recommended limit of 2,300 mg of sodium per day (4). Although about half of US adults report reducing the amount of salt they add to food, most dietary sodium comes from commercially processed and restaurant foods (5). Inadequate access to low-sodium foods makes it difficult for people to lower their sodium intake; therefore, sodium reduction strategies must extend beyond individual-level behavior change.

In 2010, the Institute of Medicine released their report, Strategies to Reduce Sodium Intake in the United States, which recommended government action to reduce sodium in the US food supply (6). The Centers for Disease Control and Prevention (CDC) responded by launching a population-level pilot program aimed at reducing the sodium content of foods served, sold, and procured across a variety of institutional settings in the United States. The first round of the Sodium Reduction in Communities Program (SRCP), from 2010 through 2013, funded state and local health departments to implement sodium reduction strategies in various settings. Strategies included implementing policies that supported sodium reduction efforts, advertising low-sodium foods to promote heart health, and adopting procurement policies to enhance sodium reduction efforts. Evaluations of the demonstration project of CDC and its awardees indicated that these strategies were a promising approach to sodium reduction, but evaluations also indicated a need for flexibility in tailoring activities to address context-specific differences among implementation sites, such as restaurants, hospitals, and schools (7–12).

## Purpose and Objectives

Recognizing the importance of incorporating evaluation findings, CDC implemented an adapted version of SRCP for the 2013 to 2016 program that included lessons learned from the initial pilot program. To build the foundation of evidence for these strategies, CDC evaluated SRCP by measuring changes in the average sodium content of foods, access to and purchase of low-sodium foods, and population intake of sodium in the pilot and in the 2013 to 2016 version. CDC conducted a comprehensive evaluation to explore the influence of SRCP strategies and associated activities on food service partners, menus, sales, and patrons of food service settings to fill a gap in the literature and to inform future work in this emerging area. Each individual SRCP awardee also conducted an internal evaluation. CDC aggregated the outcome data from the awardee evaluations to assess the effect of sodium reduction strategies in 4 domains: food service guidelines and nutrition standards, meal and menu modifications, strategies that influence the purchase of foods, and complementary consumer information activities.

## Intervention Approach

CDC funded 7 SRCP awardees in state and local health departments in 2013 and 3 additional awardees in 2014 to work on improving community support for sodium reduction and to build practice-based evidence around effective community strategies to reduce sodium consumption. Awardees represented diverse communities across the country, including states, counties, and cities from each region.

The program design consisted of 4 strategies to achieve the long-term goal of improving prevention and control of hypertension by increasing access to and availability of reduced sodium foods in the community to reduce sodium intake. Strategies included 1) developing and implementing food service guidelines and nutrition standards, 2) implementing menu or meal modifications, 3) implementing strategies that may enhance the selection and purchase of sodium-reduced foods, and 4) offering complementary venue-specific consumer information. Awardees recruited and provided technical assistance to food service partners to plan and implement activities that supported the 4 strategies. Strategies were implemented in partnering hospitals (staff and visitors), worksites (employees), independent restaurants (patrons), and congregate and distributive meal programs (ie, senior meals, early childhood education centers, prisons) for a total of 20 food service settings across all awardees. Strategies were tailored in each setting, based on goals and capacity of the partner. Awardees developed activities in collaboration with food service partners (Box).

**Box. Strategies and Activities in the Sodium Reduction in Communities Program, 2013–2016**

**Strategy 1. Develop and Implement Food Service Guidelines and Nutrition Standards**

Adopt existing nutrition standards for foods sold at food service settings (eg, US General Services Administration and Health and Human Services Sustainability Guidelines).

Develop and implement policies that set nutrition standards (eg, city or county policies for foods served in government buildings).

Develop language and implement procurement policies into vendor contracts.

Include limits for sodium in product specifications on food orders with distributors and manufacturers.

Develop healthy restaurant incentive programs and engage entities to participate.

**Strategy 2. Implement Menu and Recipe Modifications to Reduce Sodium**

Strategically plan menu cycles.

Decrease or eliminate added salt or salt-containing ingredients in a recipe.

Replace an ingredient with a low-sodium alternative in a recipe.

Modify portion sizes.

Implement standardization of recipes to measure accurate sodium content.

Eliminate the use of “free salting” (adding additional salt to recipes for flavor).

Train food service staff on culinary techniques.

**Strategy 3. Implement Strategies to Enhance Selection or Purchase of Low-sodium Foods**

Provide point of purchase nutrition information or labeling system.

Make changes to the built environment where foods are served (such as strategic placement of healthier foods).

Competitively price healthier options.

Offer taste tests or samples.

Promote low-sodium options through other initiatives (such as fresh produce as part of a culinary garden).

**Strategy 4. Offer Complementary Venue-Specific Consumer Information Activities**

Promote changes and distribute promotion materials (such as menu options, logos, table tents, menu inserts).

Train cafeteria and café operators on behavioral economics.

Collect and analyze customer satisfaction and apply feedback.

## Evaluation Methods

CDC contracted with RTI International to develop and implement a 3-year quantitative evaluation for SRCP that was based on the CDC Evaluation Framework (13). The RTI International Institutional Review Board reviewed this evaluation and determined that it is not human subject research. CDC aimed to use the evaluation to build an evidence base for community-based sodium reduction efforts by answering the overarching evaluation question, “To what extent is it possible to implement strategies in community settings to reduce the amount of sodium in foods?”

Data for our evaluation came from performance measure data, a component of awardees’ local evaluations. Awardees annually reported results of their local evaluations to CDC, including their performance measure data. On the basis of standard guidance provided by CDC, awardees selected and reported measures from a menu of options, allowing flexibility in their local evaluations according to the food service setting in which they worked, the specific activities they implemented, data availability, and interests of stakeholders. Each awardee was required to select at least 1 performance measure related to each strategy: increased availability of low-sodium foods, increased accessibility of sodium-reduced foods, increased selection and purchase of low-sodium foods, and decreased sodium intake. In this evaluation, 4 measures of program effects were examined across the strategies implemented and were most widely reported by awardees:

- Average sodium content of targeted foods or meals (n = 12)
- Number of food service organizations offering new low-sodium foods (n = 20)
- Sales of low-sodium food options (n = 5)
- Number of people purchasing or selecting low-sodium food items (n = 11)

To report performance measures, awardees developed or modified existing tools to collect baseline data at the start of the program and annually thereafter. One example of a commonly used tool is the Sodium Practices Assessment Tool, developed by one awardee and modified by others to fit their specific environment. This tool used environmental scans, pantry observations, and food service self-assessments to understand if and how sodium reduction strategies were being implemented (14). CDC aggregated data across all reporting awardees and computed the mean difference of each outcome measure at baseline and at the end of the program (3 years for 7 awardees and 2 years for 3 awardees). To evaluate the change in average sodium content, the baseline average content was subtracted from the final follow-up average content for each awardee and averaged across awardees that collected the measure. To evaluate the change in number of entities offering low-sodium foods, the total purchased low-sodium options, and the number of purchasing or selecting low-sodium foods, CDC subtracted the baseline estimate for each outcome measure from the estimate at final follow-up and summed across all awardees.

## Results

The average sodium content of targeted foods or meals decreased by 261 mg from 946 mg at baseline, to 685 mg at final follow-up in the 12 food service settings that submitted data. The reduction was largest in congregate meal programs (386 mg), followed by hospitals (223 mg) and worksites (44 mg) (Table).

**Table:** Estimated Effects of Sodium Reduction Program in Food Service Venues, Overall and by Setting, 2013–2016

Setting	No. of Awardee Settings	Baseline Intake	Final Follow-up	Change from Baseline to Follow-up
**Average sodium content of targeted foods or meals (mg)**				
Overall	12	946	685	**−**261
Congregate	5	1,484	1,098	**−**386
Hospitals	5	670	447	**−**23
Restaurants	0	NA	NA	NA
Worksites	2	287	243	**−**44
**Settings offering new low-sodium foods**				
Overall	20	0	455	+455
Congregate	6	0	91	+91
Hospitals	6	0	39	+39
Restaurants	2	0	244	+244
Worksites	6	0	81	+81
**Low-sodium food items sold**				
Overall	5	62,793	313,494	+250,701
Congregate	1	795	1,684	+889
Hospitals	2	1,353	2,623	+1,270
Restaurants	0	NA	NA	NA
Worksites	2	60,645	309,187	+248,542
**Number of people purchasing or selecting low-sodium foods**				
Overall	11	18,107	158,704	+140,597
Congregate	3	1,935	41,843	+39,908
Hospitals	1	97	665	+568
Restaurants	2	16,000	44,807	+28,807
Worksites	5	75	71,389	+71,314

SRCP activities led to an increase in the number of people with access to environments with healthy food options, including low-sodium foods. These people frequented settings where low-sodium foods were available. Across all 20 food service settings of various types, the number of organizations offering new low-sodium foods increased to an estimated 455 from a baseline of 0 organizations. The increase was largest among restaurants (244), followed by congregate meals (91), worksites (81), and hospitals (39). Combined, these organizations reached an estimated 2,029,408 people. Hospitals reached the largest number of people (1,513,755 visitors and employees), followed by worksites (366,800 employees), congregate meal programs (137,435 patrons), and restaurants (11,417 patrons).

SRCP activities also increased the sales of low-sodium foods. From baseline, low-sodium food items purchased by patrons in the 5 food service settings that reported this measure increased by 250,701 (from 62,793 items at baseline to 313,494 items at final follow-up). Most of this increase was from worksites (248,542 items).

SRCP also influenced the number of people who reduced their sodium intake through the purchase of low-sodium foods. Across 11 food service settings, an estimated 140,596 people purchased low-sodium food items compared with baseline (from 18,107 people at baseline to 158,704 people at final follow-up). The outcome was greatest in worksites (71,314 people), followed by congregate meals (39,908 people), restaurants (28,807 people), and hospitals (568 people).

## Implications for Public Health

As one of the first cross-site outcome evaluations of a community-level sodium reduction program, this evaluation’s outcomes help to build evidence for the strategies implemented. Results show that SRCP strategies increased the availability of low-sodium options by decreasing the sodium content of targeted food items, and patrons chose to purchase low-sodium items when they were available, especially in worksites. Results also suggest that when organizations implement SRCP strategies, more people have access to low-sodium foods, and the sales of low-sodium foods increase.

In addition to demonstrating the overall effects of these interventions, results also offer insight into the differential effects of food service settings that can help inform future program design. Results demonstrated the greatest potential for reach might be in hospitals (39 hospital partners reached 1,513,755 people), probably because of the large number of visitors and employees that eat in hospital cafeterias. Hospitals have an opportunity to consistently provide low-sodium food options to employees and to expose visitors to these options on a less frequent basis. Also, although 2 SRCP awardees partnered with 244 restaurants, the restaurants reached only 11,417 people, and the reach was less consistent than with other venues. Results suggest that sodium reduction efforts in worksites (71,314 people) and congregate meal settings (39,908 people) had the greatest effect on reducing sodium intake because the population remains consistent over time. Program planners should consider the tradeoff between increased reach and the consistency of that reach when identifying potential food service settings for collaboration.

The evaluation of SRCP demonstrates the potential influence of sodium reduction strategies to increase the access, availability, and purchase of low-sodium foods in a community setting and supports the need for additional community work in this emerging prevention effort. These results are essential to catalyze further action to increase low-sodium food choices and improve consumer nutrition. By partnering with commercial food service settings, SRCP targets one of the largest sources of sodium in the US food supply and addresses a major risk factor for high blood pressure.

Our study had limitations. Because the overall evaluation of SRCP relied on performance measures reported by awardees and because not all awardees were required to report the same performance measures, our evaluation was limited by incomplete data. Awardees also self-reported their data, which may have led to reporting bias, although CDC provided awardees with standard measure definitions and guidance on appropriate data sources to limit this bias. At baseline, an assessment was not completed around the extent of low-sodium offerings in partnering organizations, but all partner organizations increased low-sodium options during the program. Therefore, the measure of entities offering low-sodium foods only measures progress as a result of SRCP implementation. Additionally, SRCP could not measure sodium intake; therefore, the number of people purchasing low-sodium food items was used as a proxy. However, using this proxy limited our ability to identify duplicate counts if a patron made multiple purchases at an intervention site. A third iteration of the SRCP is being developed using lessons learned from this evaluation. The funding cycle has been increased to 5 years to provide additional time for implementation and evaluation. The evaluation will standardize performance measure reporting to strengthen the evidence of distinct sodium reduction strategies.
